# Assessment of Yeasts as Potential Probiotics: A Review of Gastrointestinal Tract Conditions and Investigation Methods

**DOI:** 10.3390/jof8040365

**Published:** 2022-04-02

**Authors:** Nadia S. Alkalbani, Tareq M. Osaili, Anas A. Al-Nabulsi, Amin N. Olaimat, Shao-Quan Liu, Nagendra P. Shah, Vasso Apostolopoulos, Mutamed M. Ayyash

**Affiliations:** 1 Department of Food Science, College of Agriculture and Veterinary Medicine, United Arab Emirates University, Al Ain P.O. Box 15551, United Arab Emirates; 950223010@uaeu.ac.ae; 2 Department Clinical Nutrition and Dietetics, University of Sharjah, Sharjah P.O. Box 27272, United Arab Emirates; tosaili@sharjah.ac.ae; 3 Department of Nutrition and Food Technology, Jordan University of Science and Technology, Irbid 22110, Jordan; anas_nabulsi@just.edu.jo; 4 Department of Clinical Nutrition and Dietetics, Faculty of Applied Medical Sciences, The Hashemite University, P. O. Box 330127, Zarqa 13133, Jordan; aminolaimat@hu.edu.jo; 5 Department of Food Science and Technology, Faculty of Science, National University of Singapore, S14 Level 5, Science Drive 2, Singapore 117542, Singapore; fstlsq@nus.edu.sg; 6 Food and Nutritional Science, School of Biological Sciences, The University of Hong Kong, Pokfulam, Hong Kong 999077, China; npshah@hku.hk; 7 Institute for Health and Sport, Victoria University, Melbourne, VIC 3030, Australia; vasso.apostolopoulos@vu.edu.au; 8 Immunology Program, Australian Institute for Musculoskeletal Science (AIMSS), Melbourne, VIC 3021, Australia

**Keywords:** autoaggregation, coaggregation, gastric, intestine, probiotics

## Abstract

Probiotics are microorganisms (including bacteria, yeasts and moulds) that confer various health benefits to the host, when consumed in sufficient amounts. Food products containing probiotics, called functional foods, have several health-promoting and therapeutic benefits. The significant role of yeasts in producing functional foods with promoted health benefits is well documented. Hence, there is considerable interest in isolating new yeasts as potential probiotics. Survival in the gastrointestinal tract (GIT), salt tolerance and adherence to epithelial cells are preconditions to classify such microorganisms as probiotics. Clear understanding of how yeasts can overcome GIT and salt stresses and the conditions that support yeasts to grow under such conditions is paramount for identifying, characterising and selecting probiotic yeast strains. This study elaborated the adaptations and mechanisms underlying the survival of probiotic yeasts under GIT and salt stresses. This study also discussed the capability of yeasts to adhere to epithelial cells (hydrophobicity and autoaggregation) and shed light on in vitro methods used to assess the probiotic characteristics of newly isolated yeasts.

## 1. Introduction

The term ‘probiotics’ refers to ‘live microorganisms that, when administered in adequate amounts, confer a health benefit on the host’ [1]. Food products containing probiotics are called functional foods and they provide health benefits, such as antihypertensive, hypoglycaemic, antioxidant [2,3,4] and immunomodulatory effects [5,6,7,8,9]. Numerous clinical studies have proven the beneficial effects of probiotics. These benefits could be conveyed either via the consumption of probiotics, as a dietary supplement, or via food products, as a probiotic vehicle [10,11,12]. Increasing awareness of the health attributes of functional foods amongst consumers has corresponded with an increased demand for the characterisation of new isolates that could be identified as novel probiotic microorganisms. As a result, there have been many medical and industrial food interests in isolating new probiotic species/strains with health-promoting benefits [13].

The International Scientific Association for Probiotics and Prebiotics published a consensus paper, regarding the characteristics of probiotics [1]. The potential probiotic microorganism must meet several criteria to be considered probiotic. The essential criteria are tolerance to the gastrointestinal tract (GIT) conditions [14] (e.g., acidic/alkaline pH, digestive enzymes and bile acids/salts), attachment to mucus and epithelial cells and sensitivity to antibiotics [15]. More importantly, the probiotic should be tested in clinical trials to be officially named a probiotic. Hill et al. [1] stated that probiotic properties are related to strain specificity, not to a particular species or genus of microorganisms.

Yeast cells are eukaryotic microorganisms ~10 times larger in size than bacteria, enabling them to act as a steric hindrance against pathogenic bacteria, thus, enhancing their prospects to be a probiotic candidate [16]. Several studies have reported the ability of new yeast isolates to resist GIT conditions, tolerate salt stress, adhere to epithelial cells and possess antimicrobial activity against various pathogens [17,18,19]. This characterisation led to research progress to employ potential probiotic yeast strains in functional foods [20,21,22,23]. However, amongst all yeast genera, only a few *Saccharomyces cerevisiae* strains have been recognised and are available commercially as probiotics for human consumption [24]. The in vitro characterisation of potential probiotic yeasts is an essential preliminary step before clinical trials. The fulfilled probiotic criteria of yeasts pave the way for conducting investigation and validation for animal models and human trials and for using yeast probiotics commercially in functional foods and for therapeutic purposes [25].

The use of probiotics in foods or dietary components provides superior health benefits to conventional food products [26]. Ogunremi et al. [27] reported using the probiotic strain of *Pichia* *kudriavzevii* OG23 to produce fermented cereal-based food, with higher antioxidant activity and various flavour compounds [27]. The combination of *S. cerevisiae* var. *boulardii* and inulin developed symbiotic yogurt. Sarwar et al. [28] reported improved product texture and an increased amount of desirable volatile compounds. In addition, the probiotic strain *S. cerevisiae* var. *boulardii* is used to produce alcohol-free beer [29].

Probiotic yeasts have also gained importance in promoting animal nutrition and health. In the past, yeast as a probiotic was employed as a feed additive, because of its rich source of fibre, protein, minerals, organic acids and B vitamins [30]. Adding viable and nonviable yeast cells to animal feed promotes health and growth [31]. Probiotic yeast (*S. cerevisiae*) has positively impacted poultry health by increasing egg production, improving feed intake and reducing plasma cholesterol [32,33]. In addition, *S. cerevisiae* has been used in ruminant feed to reduce lactate accumulation [34]. In aquaculture, stimulating the enzymatic antioxidative response of farmed fish has been reported using *Debaryomyces* *hansenii* as a dietary supplement [35].

Despite the importance of yeasts in food industries, they could contribute to the spoilage of different foods and might be pathogenic for the host. The traditional identification methods of microorganisms, based on physiological, biochemical and morphological characteristics, are insufficient and inaccurate to classify the yeast as nonpathogenic. Conventional methods are time consuming and require significant human skill. Therefore, the characterisation of yeasts at the genomic level might be decisive in distinguishing between nonpathogenic and pathogenic yeast strains. Recently, molecular methods, such as polymerase chain reaction-based techniques, mitochondrial DNA restriction analysis and chromosome electrophoretic analysis, have been effectively used in yeast strain identification [36,37]. Furthermore, rapid and reliable fragment analysis tools have been established to identify foodborne yeasts, such as random amplified polymorphic DNA, pulsed-field gel electrophoresis and restriction fragment length polymorphism [38,39,40,41,42].

The applications of yeast probiotics have been addressed in several reviews [43,44,45]. However, the assessment methods of potential probiotic characteristics of newly isolated yeasts have not been addressed. Therefore, there is a need for a comprehensive review of the assessment methods of new isolated yeasts as potential probiotics and the appropriateness of these in vitro assessment methods used. Thus, this review identified the newly isolated yeasts characterised as potential probiotics, assessed the characterisation methods and highlighted the mechanistic effects of the assessment methods on yeasts.

## 2. The Gastric Environment

Digestion is the process that degrades food substances into nutrients (e.g., phytochemicals, micronutrients and macronutrients) to release them into the bloodstream [46]. This process comprises mechanical and chemical digestion. Mechanical digestion breaks down food substances into small particles, as a prelude to effective chemical digestion [47], whereas chemical digestion is responsible for catabolising other food molecules via various digestive enzymes, to absorb them into the bloodstream [48]. Peristaltic contractions of the smooth muscle display mechanical digestion in the stomach, consisting of propulsion, churning and grinding [48]. Only tiny particles (<2 mm) can enter the duodenum [47]. The bigger particles are churned back towards the stomach for further mechanical and chemical digestion [49]. The stomach provides an environment for a fundamental part of chemical digestion. The gastric mucosa contains two glands, the so-called oxyntic and pyloric glands, associated with chemical digestion [50].

Oxyntic glands are found in the stomach. Their parietal cells produce hydrochloric acid (HCl; 160 mmol/L, pH 0.8) [51]. HCl is important to exterminate pathogenic microorganisms ingested together with foods or drinks [52] and to activate pepsin from the zymogen pepsinogen, which is secreted by the chief cells of oxyntic glands [50]. Another intrinsic function of HCl is protein denaturation, to facilitate enzymatic digestion by pepsin. Pepsin breaks down the peptide bonds of proteins at the optimal pH of 2.0–3.0 into individual amino acids, which are released into the bloodstream [47,50]. The pyloric glands are located in the stomach antrum [53]. They form the hormone gastrin secreted by their enteroendocrine G-cells, which act to induce the creation of HCl [51,53]. In addition, mucus secreted by mucous cells of the pyloric glands plays a significant role in protecting the gastric surface from the acidic medium in the stomach [54].

### 2.1. Tolerance to Gastric Conditions

Several studies have defined the growth inhibition of microorganisms subjected to GIT conditions [55,56,57]. Acids can diffuse passively through the cell membrane and, thus, access the cytoplasm, dissociating into charged derivatives and protons [58]. Proton accumulation in the intracellular cytoplasm may decrease the intracellular pH and, subsequently, affect the transmembrane pH gradient [59], which contributes to the proton-motive force and minimises the amount of energy obtainable for cellular growth [60,61]. Furthermore, internal acidification reduces the inhibition of the action of acid-sensitive microorganisms, but extremely low internal pH induces damage to DNA and enzymes and denatures proteins, resulting in cell death [62,63]. Besides accumulating dissociated organic acids in the cytoplasm, this condition has a destructive effect on cellular physiology [64]. Gastric stress experiments with different potential probiotic yeasts are summarised in Table S1 (pH 2.0, 0.0133 g/L pepsin, 2.5 h and 37 °C). The survival rate of *Metschnikowia pulcherrima* isolated from fermented table olives reached 96.4% [65]. This survival percentage was relatively lower in *Candida adriatica* collected from olive oil (23.4%) [66].

**Figure 1:**
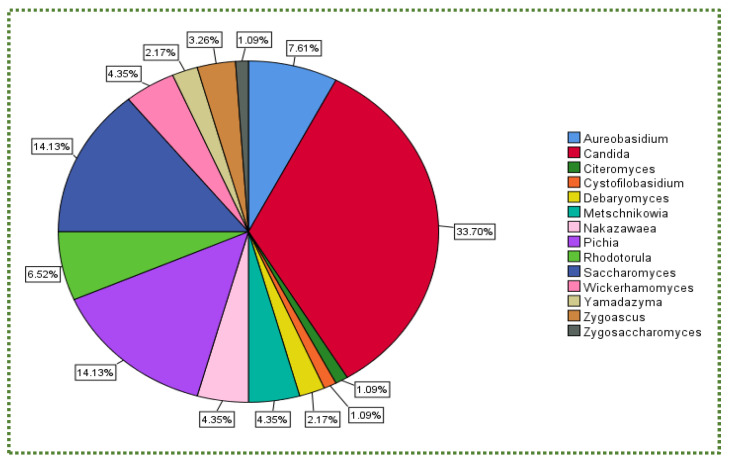
Total number (%) of isolated yeast genera under gastrointestinal conditions.

**Figure 2:**
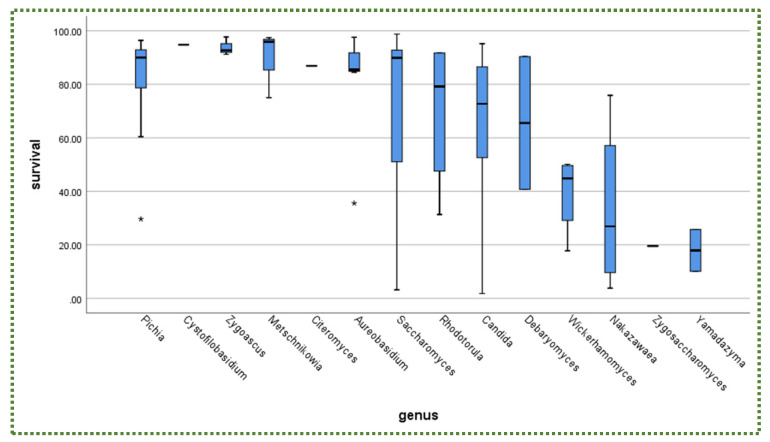
Survival rate (%) of yeasts under gastric conditions in descending order according to genera. * is outliners.

Figure 1 illustrates the percentage of the total number of isolated yeast genera subjected to gastric and intestinal conditions. The boxplot chart in Figure 2 displays the percent survival rate under gastric conditions. Although *Candida* did not exhibit the highest survival rate (Figure 2), it was the most frequently isolated genus at 34%, followed by *Pichia* and *Saccharomyces* (14% for each) and *Aureobasidium* (7.6%; Figure 1).

### 2.2. Assessment Methods of Gastric Tolerance

Three factors must be considered when adopting such a method for screening GIT stress tolerance. These factors are derived from the digestion process in vivo: (1) diffusion of gastrointestinal fluids into the food matrix, (2) synchronisation of mechanical digestion with chemical digestion and (3) sequence of enzymatic degradation. Therefore, time is a decisive parameter.

The significant challenges in the stomach are the extreme acidity, pH from 2.0 to 3.0, and the presence of digestive enzymes, such as pepsin [67]. This condition inhibits most microorganisms, including yeasts [68]. To qualify as probiotics, yeast strains have to survive the gastric conditions and reach the gut alive, where they will exert their function [69]. Some microorganisms, including yeasts, possess the capability to survive and grow in an acidic medium. The survival of selected yeast strains against digestion conditions is usually evaluated in vitro, in a gastric-like environment, where simulated gastric juice is prepared in a buffer solution at a low pH level (preferably pH 2.0) in the presence of pepsin, for given time intervals at 37 °C [70]. This method aims to quantify strain viability after being subjected to gastric juice. However, many researchers [18,71,72] used only acidic pH without adding pepsin to evaluate pH tolerance. They overlooked assessing gastric tolerance as a precondition to consider such microorganisms as probiotics, leading to inaccurate outcomes.

For all studies in this review (Table S1), a buffer solution consisting of NaCl, KH_2_PO_4_, CaCl_2_ and KCl was used. Cells of each yeast strain were resuspended in gastric fluid and incubated for 2.5 h at 37 °C [65,66,70,73,74]. Given the importance of mechanical movement, no studies (Table S1) mentioned the application of mechanical movement while assessing the gastric tolerance of potential yeast probiotics as a part of their assays, which could result in an imprecise assessment.

### 2.3. Mechanisms of Gastric Tolerance

The underlying mechanism for surviving yeast under low pH is modifying the yeast cell wall [75,76]. Strong inorganic acids, such as HCl, in the stomach and yeast cells adjusted to low pH comprise a mechanism that activates the cell wall integrity pathway [77,78]. It depends on the transmission of the signals of stressed walls to Rho1 GTPase, leading to the formation of various carbohydrate polymers used for remodelling the cell wall [79]. Another mechanism for resisting the strong inorganic acid is a general stress response pathway, an essential response against any conditional alteration, where the conductance of the protein kinase C pathway is fundamental [76].

In addition, under low pH, the temporal inefficiency of the glucose-sensing pathway in yeast and growth inhibition are the main causatives of the activity reduction in protein kinase A, thereby liberating general stress responses and driving to remodel cell gene expression, to adjust to the low-pH condition [76,78,80]. Furthermore, calcium metabolism could affect yeast responses to low external pH [81], in which the deletion of either Mid1p or Cch1pm, as calcium channels, is vital to yeast cells when subjected to inorganic acid stress [82,83].

Adjusting the membrane lipid composition in *Candida glabrata* by mediator subunits led to increases in acid tolerance, up to pH 2.0, indicating the significant role of lipid homoeostasis [84]. Fletcher et al. [85] proved that acid tolerance could be acquired by altering sterol composition and reducing iron uptake, such as in *S. cerevisiae*, as its acidic tolerance developed to pH 2.8. In line with previous studies, the use of pepsin and low pH (2.0) is recommended to mimic human gastric conditions and, thus, obtain an accurate assessment when examining new isolates in gastric juice.

## 3. Intestinal Conditions

The small intestine is associated with pancreatic enzymes and bile released from the liver to continue digestion. The secretions of the pancreas and duodenum mix with digesta and chyme [47]. As peristalsis occurs, mechanical digestion goes on, slightly. The small intestine environment is neutral to slight alkalinity because of the bicarbonate produced by the pancreas. It allows the digestive enzymes secreted by the duodenum and pancreas (e.g., pancreatic amylase, pancreatic lipase and trypsinogen) to act optimally at pH 6–7 [86,87]. Pancreatic amylase is responsible for hydrolysing starch into maltose and maltotriose [88]. At the same time, pancreatic lipase is associated with a coenzyme, named colipase, for hydrolysing triglycerides to produce diacylglycerols and monoacylglycerols [88,89]. Trypsinogen is a zymogen of trypsin. It is converted to its active form (trypsin) by enterokinase, and trypsin converts other pancreatic zymogens to their active forms [90].

Pancreatic zymogens transfer to the common bile duct, creating the ampulla of Vater, and empty its contents into the duodenum, where pancreatic zymogen activation occurs. Liver cells form bile, which is stored in the gallbladder and carried by the common bile duct [91]. Bile contains a mixture of bile salts, fatty acids, cholesterol, electrolytes and bilirubin that make the total solution basic with an average pH of 8.2 [92,93]. Bile salts and acids pass to the small intestine, where they function as detergents for waste products from the blood and are critical for breaking down fat into fatty acids [94].

### 3.1. Tolerance to Intestinal Conditions

The major obstacles to yeast survival are high bile salt and organic acid concentrations. Moreover, Pais et al. [95] highlighted that pancreatic and hydrolytic enzymes, secondary metabolic products of the gut microbiome and epithelial brush border in the small intestine, might destroy microorganisms, including yeasts. Bile salts are generally created from cholesterol in the liver and secreted into the intestine to contribute to the digestive process [96]. Previously, Urdaneta and Casadesús [97] stated that bile salts have detergent properties, and as a result, they can be toxic to the GIT microbiota. However, some microorganisms resist bile salts and hydrolytic enzymes [98].

Alkaline stress is another challenge that inevitably exposes yeast strains in the intestinal tract. For example, *S. cerevisiae* grows in acidic pH better than in alkaline conditions [77]. These strains do not proliferate when pH exceeds 8.0–8.2 [99]. Indeed, *Schizosaccharomyces pombe* growth was inhibited, even at neutral pH [100]. Nevertheless, some yeast species, such as *Yarrowia lipolytica*, are resistant to alkaline conditions up to pH 10–11 [101,102]. Previous studies confirmed that pH changes lead to upregulated and downregulated gene expression in *S. cerevisiae* [103,104,105].

The active transporters of the plasma membrane are activated by the permanent proton gradient between extracellular and intracellular media, which is protected by respective fungal orthologs, for instance, in *S. cerevisiae* by Pma1p H^+^-ATPase [106]. The importance of this gradient comes from its functional uptake of several vital compounds [61]. Acidic stress affects it slightly, but alkaline stress damages it [106]. When cells cannot neutralise a circumambient alkaline medium, microorganisms suffer from starvation for nutrients, such as glucose and phosphate [107,108]. In addition, extreme external pH reduces the ionisation of primary transition metals, leading to starvation for these metals (e.g., iron and copper) [109].

Table S2 summarises various yeast strains undergoing in vitro intestinal conditions (pH 8, 0.1 g/L pancreatin, 3.0 g/L bile salts, 3.5 h and 37 °C). Bonatsou et al. [74] tested the response of *Rhodotorula diobovatum* isolated from Greek black olives, which displayed only a 3.83% survival rate. Other species isolated from the same source (*Rhodotorula mucilaginosa*) reached 53.40%. The differences in the survival rate were species- and strain-dependent.

**Figure 3:**
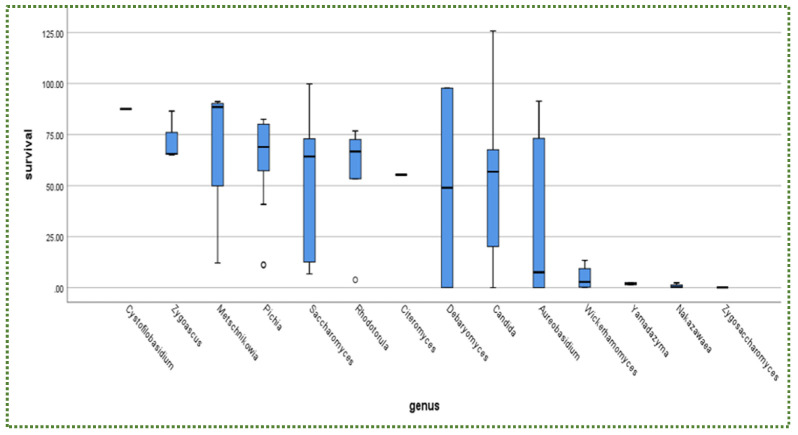
Survival rate (%) of yeasts under intestinal conditions. o = outliners.

The percentage of the total number of isolated yeast genera undergoing intestinal conditions is presented in Figure 1 (pH 8, 0.1 g/L pancreatin, 3.0 g/L bile salts, 3.5 h and 37 °C). Figure 3 shows that *Cystofilobasidium* achieved the highest survival rate, whereas *Zygoascus* occupied the second rank, followed by *Metschnikowia* and *Pichai*, which, relatively, retracted compared to their position in gastric tolerance.

### 3.2. Assessment Methods of Intestinal Tolerance

Intestinal tolerance is a prerequisite to consider yeast as a probiotic, in addition to its survival under notable pH and temperature variations, through their passage from the stomach to the small intestine [110]. The assessment of intestinal tolerance of the potential yeast probiotic is mainly performed based on a similar concept to the in vitro gastric condition test. The optimum concentration of the bile in the human gut environment and the actual human temperature range are 0.3–0.6% and 36.5–37.5 °C, respectively [111]. Thus, isolated yeasts are commonly incubated at 37 °C for ~3.5 h after resuspension in an intestinal-like fluid, containing pancreatin and bile salts at pH 8.0. Both sodium phosphate dibasic heptahydrate and sodium chloride were used to make a buffer solution for experiments of intestinal tolerance in recently reviewed studies.

**Figure 4:**
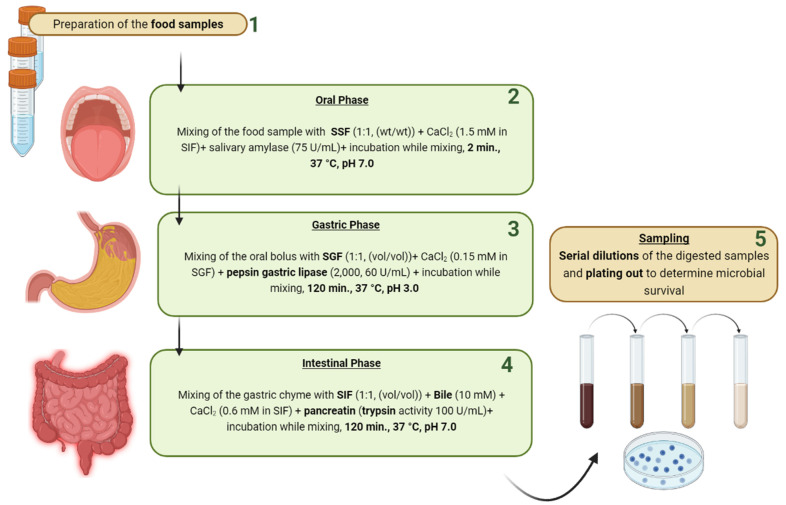
Schematic in vitro digestion method. SSF, simulated salivary fluid; SGF, simulated gastric fluid; SIF, simulated intestinal fluid.

The INFOGEST in vitro digestion model (Figure 4) is widely applied in food research. This method includes three successive stages that simulate the digestion process in the upper GIT in vivo. The modality to implement INFOGEST is based on pH values, enzyme activity and the ionic strength of electrolyte solutions used in oral, gastric and intestinal phases. Furthermore, the digestion durations are 2.0, 120 and 120 min, respectively, under agitation status [112]. Thus far, according to the three factors mentioned in Section 2.2, the INFOGEST static model is considered a more reliable method to evaluate the GIT stress tolerance for microorganisms because it is almost the most simulated in vivo GIT. However, to the best of the authors’ knowledge, up to now, there is no application of the INFOGEST assay on the assessment of the GIT stress tolerance of yeast probiotics. By contrast, screening for this essential probiotic criterion of a potential probiotic bacterium by the INFOGEST model has been applied by many researchers [113,114,115].

### 3.3. Mechanisms of Intestinal Tolerance

According to FAO/WHO [116], resistance to bile acids/salts is a precondition for probiotic yeast to survive in the small intestine through the passage in the GIT. Bile salts have strong toxic effects on the cellular membrane of microorganisms [117], in terms of their fluidity, charge and hydrophobicity, perturbing cellular homoeostasis and motive oxidative stress [118]. However, several microorganisms can overcome the toxic effects of bile salts by employing bile salt hydrolases (BSH) of the intestinal microbiome, minimising the toxic influence of conjugated bile [119]. The mechanism of bile resistance in yeast is not entirely understood [120]. Despite that, bile salt resistance in probiotic yeasts has been shown by several studies [18,121]. Recently, a BSH, an enzyme responsible for hydrolysing bile salts, was investigated in vitro and detected in *Saccharomyces* *boulardii* [122], which might contribute to understanding yeast bile resistance.

Numerous mechanisms have been suggested to explain yeast resistance regarding yeast alkaline tolerance. One of them occurs in *S. cerevisiae*, where yeast plasma membrane Ena1 P-type *ATPase* acts on the cell’s efflux K^+^ and Na^+^ cations, counteracting the internal alkaline medium [106,123]. Another mechanism in *S. cerevisiae*, the protein kinase A pathway, is inhibited under alkaline stress, leading to the remodelling of *Msn2* and *Msn4* gene expression of stress-responsive transcriptional activators, to offset environmental alkalisation [124]. Moreover, Casamayor et al. [107] documented that *S. cerevisiae* resorts to glycogen *mobilisation* to respond to alkaline pH stress, to compromise glucose uptake. The vacuolar H^+^-ATPase enzyme is also essential to regulate intracellular pH in yeast cells [125,126]. Another strategy is adopted by *Y.* *lipolytica* to cope with alkaline stress, using polyphosphate storage molecules. The elevated pH of the cytosol drives polyphosphate hydrolysis, which compensates for the phosphate storage and restores the proper pH of the intracellular medium [101,127]. Simulating the intestinal fluid in the presence of pancreatin bile salts, preparing them at a similar concertation to the natural digestive system and setting a pH range from 7.8 to 8.0 are keys to an accurate appraisal of new isolates against intestinal juice.

## 4. Salt Conditions

In several food processes, probiotic cells are constantly subjected to environmental stresses, including excessive salinity, which forms hyperosmotic stress (salt stress). Probiotic yeasts could be exposed to hyperosmotic stress during the food production process, such as in the production of fermented food and certain cheese varieties [111,128].

In recent years, there have been a variety of food matrices targeted as probiotic vehicles containing significant amounts of NaCl [129]. For instance, a high-brine solution has been used as a flavour improver and preservative agent [130]. Fermentation of black olives occurs in 80–100 g/L brine [131], whereas the salt concentration in soy sauce reaches up to 18% [132] or higher. During the fermentation process, salt can repress the growth of moulds and some yeasts, the main causative agents of food spoilage, and can suppress the growth of specific foodborne pathogens, such as *Listeria monocytogenes* and *Staphylococcus aureus* [133,134]. Further, salt preservative properties are associated with shelf-life extension, flavour improvement and fermented food products [130,135].

### 4.1. Salt Stress

Salt tolerance can be a critical feature for selected yeast strains, thus, partially qualifying them as probiotics in traditional food fermentation or processing. However, an elevated saline environment can damage enzyme structure, suppress metabolic enzyme activity and retard fermentation [136]. Moreover, Heinisch and Rodicio [137] documented that high salinity may drive cell plasmolysis, and intracellular water molecules could diffuse out of the cells. Hence, owing to the lack of timely and efficient responses, high-salt conditions could cause growth inhibition or yeast death [138]. However, some yeast strains can survive and grow under such an environment, relying on strain-specific abilities to detect and respond to salt stress [139]. Table S3 presents the assessed non-inhibitory concentration (NIC) and minimum inhibitory concentration (MIC) values (g/L) measured under NaCl conditions for some yeast strains. *S. cerevisiae* isolated from Kalamata table olive fermentation and subjected to NaCl tolerance test at pH 3.5, 5.0 and 6.5 showed MIC values of 146.73, 174.8 and 143.8 g/L, whereas the same source was used to collect *Zygoascus hellenicus* and demonstrated MIC values of 125, 147 and 129 g/L.

**Figure 5:**
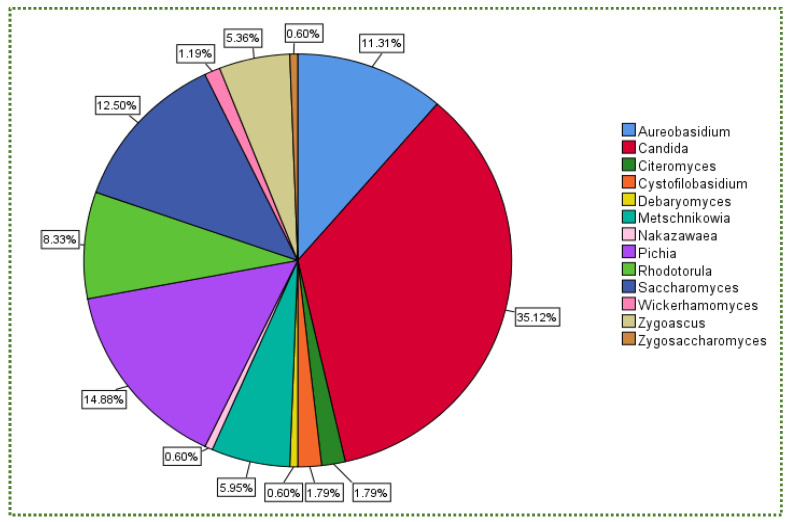
Total number (%) of isolated yeast genera after NaCl stress.

**Figure 6:**
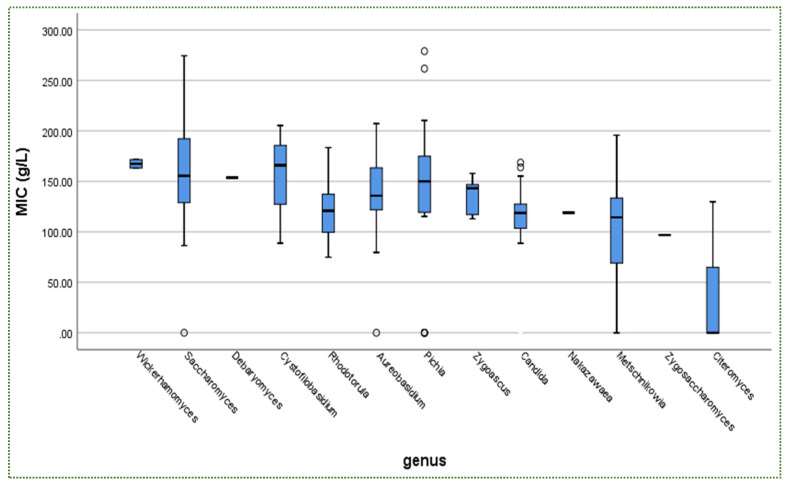
MIC (g/L) of yeasts under NaCl conditions in descending order according to genera. o = outliners.

From the same data, *Candida* was the most isolated genus (35%) amongst the 13 genera (Figure 5). Although it did not achieve the highest mean MIC value (Figure 6), which was recorded for *Wickerhamomyces*, it was not halophilic.

### 4.2. Assessment Methods of Salt Tolerance

Lately, salinity tolerance test was carried out in vitro by adding yeast suspensions to appropriate broth (e.g., yeast malt broth), supplemented with different NaCl concentrations (e.g., 0–250 g/L) at various pH values (e.g., 3.5, 5.5 and 6.5). The growth of the strains was measured as optical density (OD) at 420 nm, in an automatic spectrophotometer for a specific period (e.g., 12 h for a fixed interval, such as 7 days). The NIC and MIC at different NaCl/pH values for each period were calculated by comparing the area of the curves under OD/time for the control, where salt was absent to the area of the curves where salt at different pH values was used [65,70]. Because salt is used in many processed foods, the inaccuracy in assessing its tolerance may suppress the growth of putative probiotic strains in the food product. Accordingly, yeast strains subjected to salt stress for short periods (2 h) may not give correct information, regarding the ability of the strains to tolerate salt (Bonatsou et al.) [74].

### 4.3. Mechanisms of Salt Tolerance

Hyperosmotic stress induces water efflux, resulting in cell shrinkage, excessive cellular solute concentrations and abolished cell turgor pressure [138,140]. Microorganisms counteract such influences by coping with various energetic mechanisms that modify cellular energy homoeostasis and reduce microbial growth [134]. Cells adjust their intracellular osmotic pressure by producing osmolytes, which are small organic solutes (such as glycerol and trehalose) that help in retaining intracellular water [141,142].

Hyperosmotic stress tolerance in yeasts is adopted via the high osmolarity glycerol (HOG) pathway, where mitogen-induced protein kinase is activated by leveraging two cell-membrane-bound receptors [140,143]. More specifically, sho1p and sln1p reveal osmotic alterations, leading to the stimulation of HOG pathway genes [106,144] and resulting in downstream triggered genes related to salt stress resistance [145]. When conducting a salt tolerance test to evaluate novel probiotic yeasts, it is recommended to apply different NaCl concentrations at various pH values to estimate each environment in fermented food matrices and targeted food processing protocols. The results could indicate the strains that may survive and dominate during fermentation.

## 5. Autoaggregation

Autoaggregation is associated with promoting colonisation in the human intestine, immunomodulation of the colonic mucosa and prevention of pathogenic infections [146,147]. Intestinal epithelial cells are covered by the mucosal glycocalyx layer that mainly contains electrolytes, immunoglobulins, glycolipids and glycosylated proteins (mucins) with sugar residues [148]. Adhesion property is gained by particular cell surface proteins, named ‘flocculins’ or ‘adhesions’, on the yeast cell surface that bind sugar residues, as on mucins in epithelial cells or certain amino acids on the surface of the other cells [149].

The autoaggregation property is represented as the ability of yeast strains to self-aggregate and produce flocs as a survival response, which extends a competitive advantage over other microorganisms, including enteric bacterial pathogens in severe conditions, such as human GIT [25]. This ability is an essential property of selected probiotics. Fernandez-Pacheco Rodríguez et al. [150] noted that probiotics should adequately adhere to epithelial cells to colonise the intestinal mucosa and exert their functional effects. In addition, the creation of cellular aggregates expands cell protection in an adverse environment [151]. Because yeast cells are larger and heavier than bacteria, they sediment faster and in a higher amount [152].

**Figure 7:**
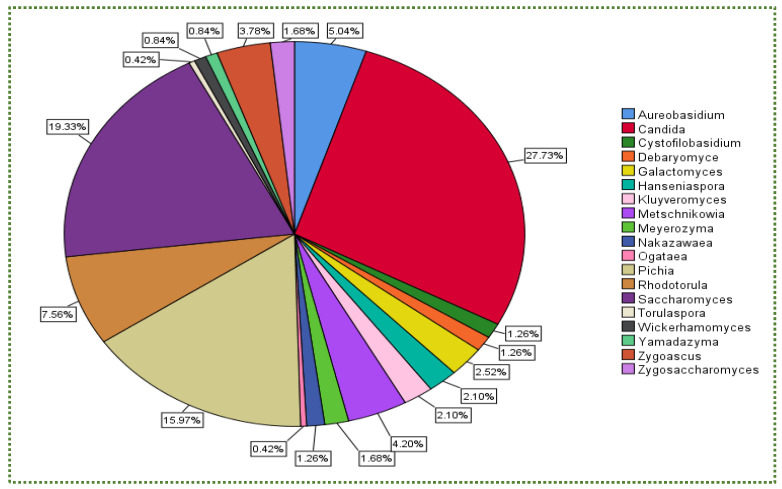
Total number (%) of isolated yeast genera that underwent the autoaggregation test.

**Figure 8:**
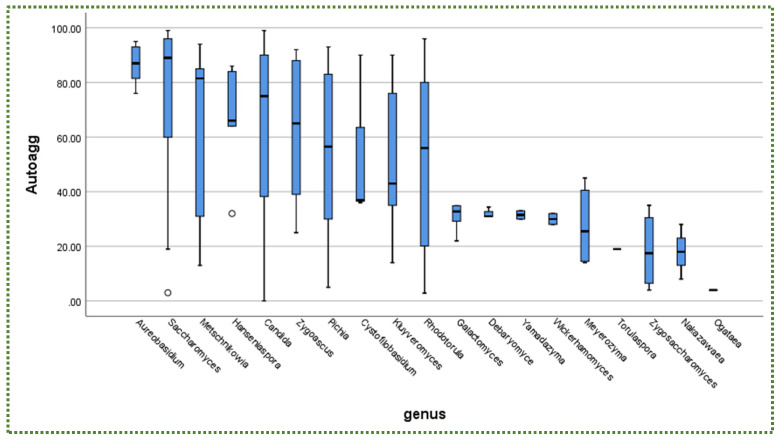
Autoaggregation (%) in descending order according to yeast genera. o = outliners.

The outlines of the autoaggregation capacity of the selected yeast strains, measured in different periods, are shown in Table S4. *Candida molendinolei* isolated from Kalamata table olive fermentation exhibited 42%, 48% and 93% autoaggregation percentages after 2, 4 and 24 h of incubation. After 4–24 h of incubation, the yeast strains continuously increased their capability to make cellular aggregates. *Candida tropicalis* is another *Candida* species, collected from fermented Portuguese table olives, demonstrating ~86% autoaggregation. *Nakazawaea wickerhamii* isolated from olive oil displayed only 18%. This variance showed that the autoaggregation capability is substantially strain-dependent [153]. In another perspective, the most frequently isolated genus amongst yeast genera was *Candida* with 27.7%, followed by *Saccharomyces* and *Pichia* with 19.3% and 16%, respectively (Figure 7), whereas the highest autoaggregation percentage was exhibited by *Aureobasidium* and *Saccharomyces* (Figure 8).

### 5.1. Assessment Methods of Autoaggregation

Generally, the autoaggregation percentage of selected yeast strains proceeds in vitro via the resuspension of the suspended strains in phosphate-buffered saline (PBS) or NaCl solution (mostly 0.9%, *w*/*v*), and the test is carried out in a disposable plastic cuvette. The absorbance is measured at 600 nm, using an automatic spectrophotometer at different time intervals, without shaking the cell suspensions [65,73]. Yeast strains isolated by Gut et al. [25] and Fernandez-Pacheco Rodríguez et al. [150] had undergone autoaggregation evaluation for only 2 h or 30 min, respectively. Quantifying the autoaggregate capacity of yeast strains under a short time could not reflect their actual capacity. In vitro, the median transit time through the gut is 28.7 h [154,155], and the mean short intestinal transit time is 4.2 h [156], where the autoaggregation phenomenon is expected to activate its function.

### 5.2. Mode of Action of Autoaggregation

Autoaggregation (or, to be exact, ‘flocculation’) in yeasts is a complex phenomenon that takes place predominantly upon sugar depletion throughout the plated exponential or stationary phase [157]. In this circumstance, autoaggregation is influenced by the differences in cell wall composition, the presence and morphological type of cell appendages and the protruding macromolecules from the cell wall [158].

Di Gianvito et al. [159] demonstrated that flocculation occurs in two main phases: equal cell–cell adhesion is formed on glycan–glycan interaction and the glycan–lectin binding. First, glycan–glycan interaction is fixed by specific proteins, known as flocculins, zymolectins, adhesins or lectins (lectin-like theory) [160]. Then, residual mannose in the cell wall is bound by any proteins that protrude from the cell surface [161]. Ca^2+^ ions in the environment are significant because of their contribution to sugar binding and maintaining the correct flocculin conformation [160,162]. To gain the correct autoaggregation result, it is advisable to perform the test at gradual time intervals and suspend the strains in a saline buffer solution at the simulated NaCl concentration in the human GIT.

## 6. Hydrophobicity

Another property of potential probiotic strains is cell surface hydrophobicity. As in autoaggregation, hydrophobicity is crucial in reflecting the ability of probiotic adhesion and colonisation in the epithelial cells of the human GIT, where they may extend prophylactic and therapeutic impacts [163], resulting in the prevention of pathogen colonisation by their interactions [164]. The tendency of microorganisms to adhesion can increase according to the surface type [165]. Thus, cells with high hydrophobic properties attach more strongly to hydrophobic surfaces [166]. Surface hydrophobicity in mammals, including humans, is very high on the top of the gastric mucosa and the colon [167]. This hydrophobic property is attributed to the surface-active phospholipid layer that lines the mucus top that coats the epithelium [168]. Van Tassell and Miller [169] stated that, initially, the reversible and weak physical binding between probiotics and mucosa has occurred through nonspecific contact by hydrophobicity and spatial recognition. Subsequently, stable binding has been established between probiotic adhesins, usually surface-anchored proteins and complementary receptors in the mucus or intestinal epithelial cells, effectively colonising the GIT [169].

The correlation between the microbial ability to colonise the GIT and hydrophobicity has been studied in vivo and has been debated widely [1,170,171]. Nevertheless, this is not a compulsory characteristic, as yeasts are commonly excreted in the faeces because of competition with the GIT microbiota [172]. Therefore, probiotic yeasts should be regularly consumed to conserve appropriate balance in the host track, considering that the probiotic impact is dose-dependent [15].

**Figure 9:**
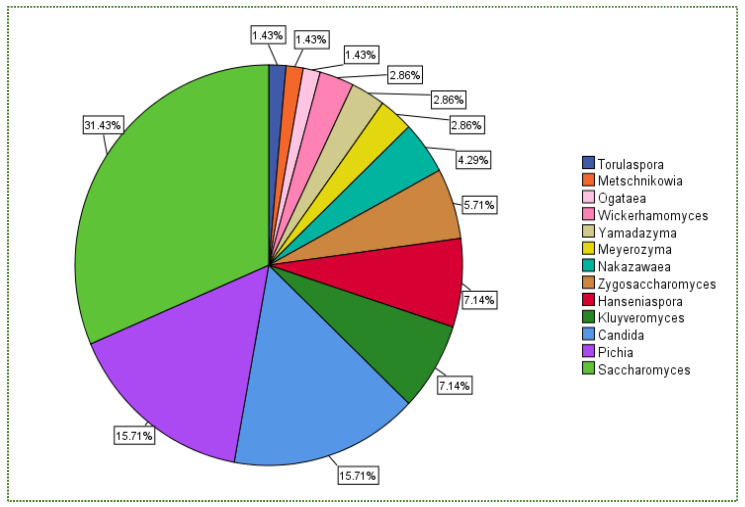
Total number (%) of isolated yeast genera measured as the hydrophobicity ability towards n-hexadecane.

**Figure 10:**
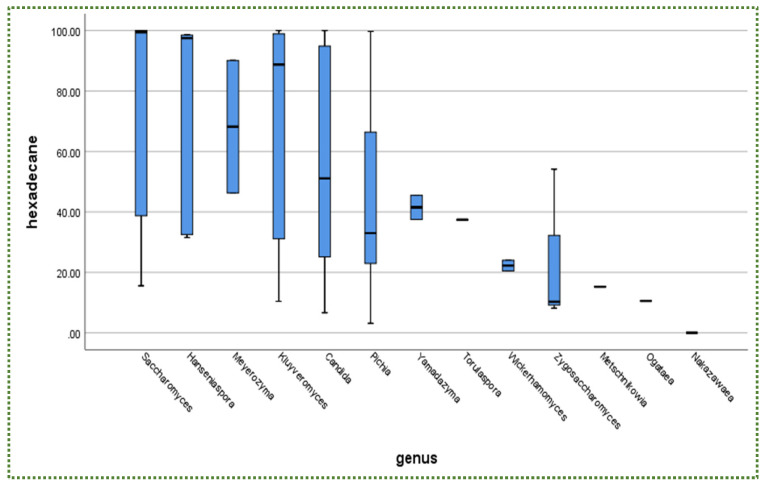
Hydrophobicity (%) towards n-hexadecane in descending order according to yeast genera.

The hydrophobicity of the studied yeast strains towards n-hexadecane is presented in Table S5. Interestingly, all strains isolated from fermented foods by Menezes et al. [173] resulted in >90% hydrophobicity, confirming that fermented foods could serve as carriers for probiotic microorganisms [174], including yeast. From another viewpoint, *Saccharomyces* is the most often isolated genus (Figure 9), followed by *Pichia* and *Candida*, regarding the ratio of the isolated yeast genus. However, these latter two genera hold the fifth (55.47%) and sixth (45.35%) positions, respectively, in terms of their ability towards hydrophobicity (Figure 10).

### 6.1. Assessment Methods of Hydrophobicity

The technique often used in vitro to assess hydrophobicity is measuring microbial interaction with hydrocarbons, such as n-hexadecane. This method is based on the microorganism’s affinity to a nonpolar solvent (e.g., n-hexadecane) [175]. Initially, strains are resuspended in buffer (e.g., 0.1 M KNO_3_ or 0.1 M PBS, pH 7). After measuring OD_600 nm_ as the initial absorbance, the addition of n-hexadecane, xylene and octane to independent samples is conducted. After incubation at 37 °C for 60 min, the absorbance of the aqueous phase is measured [22,66,150].

Gut et al. [25], Zullo and Ciafardini [66] and Menezes et al. [173] used only n-hexadecane to examine the capacity of putative yeast probiotics, which may give unsatisfactory results. By contrast, microcell adhesion to the intestinal epithelium is linked to several factors, such as lectins, passive forces, lipoteichoic acid and hydrophobic forces [176,177]. Therefore, the ability to be hydrophobic is not determined by the presence of a particular hydrocarbon solvent. For example, Linder [178] stated that the self-assembly of filamentous fungi refers to hydrophobins and surface-active proteins, whereas Hazen and Hazen [179] found that the main factor of the hydrophobic feature in *Candida albicans* is the surface fibrils. Thus, the greater the variety of nonpolar solvents used to evaluate hydrophobicity, the more accurate the results.

### 6.2. Mechanisms of Hydrophobicity

Hydrophobicity is a physicochemical characteristic of the microbial cell surface, where a cell surface protein and lipoteichoic acids mediate a nonspecific interaction between microbial and host cells [180,181]. Lara-Hidalgo et al. [182] reported that strains with >40% hydrophobicity are hydrophobic. In this respect, hydrocarbons of the host cell will be bound by those strains and will be shifted from the aqueous to the organic phase of the environment [183]. Hydrophobicity has been attributed to complex interactions between hydrophobic and hydrophilic (negatively and positively charged) components on the microbial cell surface. To evaluate the cell surface hydrophobicity of newly isolated yeasts, different hydrocarbons, such as n-hexadecane, xylene and octane, should be used to mimic the adhesion efficiency of the intestinal epithelium.

## 7. Insights and Future Recommendations

Studying gastrointestinal and salt tolerance in vitro has revealed the possibility of yeast cells surviving under harsh conditions. Thorough knowledge of the mode of action that supports the adaptation of such gastrointestinal and salt stresses can promote yeasts as probiotics. Simulating the required conditions in the food industry and mimicking the biological processes to which yeasts in the human body are exposed are the decisive keys to accurately assess the probiotic properties of potential yeast isolates.

In addition, there is no relationship between yeast availability (Figure 1 and Figure 4) and its survivability (Figure 2, Figure 3 and Figure 5) under stresses and activities (autoaggregation and hydrophobicity), as reviewed by this study. This review also showed that *Pichia* and *Cystofilobasidium* achieved the highest survival rate under GIT stresses, indicating that it is valuable to focus on their molecular and genetic mechanisms to advance to in vivo trials, to exploit them in the production of functional foods and probiotic dietary supplements. This review included mainly the more robust probiotic properties (GIT and salt tolerance) presented by fermented food compared to unfermented food. In addition, the higher capability of cell surface properties, autoaggregation and hydrophobicity were demonstrated by isolates that achieved higher survivability under GIT stress (positive correlation).

The differences between bacteria and yeasts dictate the necessity to determine the mechanisms adopted by yeast probiotics to overcome extreme conditions. In this context, yeast resistance to bile acids/salts requires further studies. Further investigation is recommended to evaluate potential yeast probiotics in the food industry, such as survivability under heat stress and the ability to produce exopolysaccharides and safety aspects, including antibiotic sensitivity and the absence of virulence genes.

## Data Availability

Not applicable.

## Supplementary Materials

The following supporting information can be downloaded at: https://www.mdpi.com/article/10.3390/jof8040365/s1, Table S1: The survivability of selected yeast strains undergo in-vitro gastric conditions. Table S2: The survivability of selected yeast strains undergoes in-vitro intestinal conditions. Table S3: Evaluated of NIC (non-inhibitory concentration) and MIC (minimum inhibitory concentration) values (g/L) measured under salt (NaCl) for the yeast strains. Table S4: The percentage auto-aggregation capacity of the selected yeast strains, incubated at 37C and measured at different time intervals. Table S5: The percentage of hydrophobicity capacity of the selected yeast strains, incubated at 37C and measured at different time intervals.
