# Supplementary material for: Assessment of Yeasts as Potential Probiotics: A Review of Gastrointestinal Tract Conditions and Investigation Methods
Source: J Fungi (Basel). 2022 Apr 2;8(4):365. doi: 10.3390/jof8040365 (PMC9027893; doi:10.3390/jof8040365)
Supplement: Supplementary file 1 [file jof-08-00365-s001.zip › jof-1629168-supplementary/jof-1629168-sm-conversion.pdf]

Assessment of Yeasts as Potential Probiotics: A Review of Gastrointestinal Tract Conditions and Investigation Methods

Supplementary Materials

Nadia S. Alkalbani [1], Tareq M. Osaili [2,3], Anas A. Al-Nabulsi [3], Amin N. Olaimat [4], Shao-Quan Liu [5], Nagendra P. Shah [6], Vasso Apostolopoulos [7,8] and Mutamed M. Ayyash [1,*]

[1] Department of Food Science, College of Agriculture and Veterinary Medicine, United Arab Emirates University, Al Ain 15551, United Arab Emirates; 950223010@uaeu.ac.ae
[2] Department Clinical Nutrition and Dietetics, University of Sharjah, Sharjah 27272, United Arab Emirates; tosaili@sharjah.ac.ae
[3] Department of Nutrition and Food Technology, Jordan University of Science and Technology, Irbid 22110, Jordan; anas_nabulsi@just.edu.jo
[4] Department of Clinical Nutrition and Dietetics, Faculty of Applied Medical Sciences, The Hashemite University, P. O. Box 330127, Zarqa 13133, Jordan; aminolaimat@hu.edu.jo
[5] Department of Food Science and Technology, Faculty of Science, National University of Singapore,
S14 Level 5, Science Drive 2, Singapore 117542, Singapore; fstlsq@nus.edu.sg
[6] Food and Nutritional Science, School of Biological Sciences, The University of Hong Kong,
Pokfulam, Hong Kong 999077 , China; npshah@hku.hk
[7] Institute for Health and Sport, Victoria University, Melbourne, VIC 3030, Australia; vasso.apostolopoulos@vu.edu.au
[8] Immunology Program, Australian Institute for Musculoskeletal Science (AIMSS), Melbourne, VIC 3021, Australia
* Correspondence: mutamed.ayyash@uaeu.ac.ae

Table S1. The survivability of selected yeast strains undergo in-vitro gastric conditions

Genus	Species	Source	Survival Mean	Minimum	Maximum	Count	Reference
Aureobasidium	*Aureobasidium pullulans*	Greek black olives	35.55	35.55	35.55	1	(S Bonatsou et al., 2015)
		table olive fermentation	89.38	84.5	97.6	6	(Stamatoula Bonatsou et al., 2018)
Candida	*Candida adriatica*	Olive Oil	23.4	16.6	30.2	2	(Zullo & Ciafardini, 2019)
	Candida boidinii	fermented black olive	29.79	4.19	55.38	2	(Porru et al., 2018)
		fermented Portugalian table olives	16.16	16.16	16.16	1	(Oliveira et al., 2017)
		table olive fermentation	90.12	85.5	95.2	6	(Stamatoula Bonatsou et al., 2018)
	Candida diddensiae	fermented black olive	65.67	64.94	66.4	2	(Porru et al., 2018)
		Olive Oil	2.4	1.78	3.01	2	(Zullo & Ciafardini, 2019)
		table olive fermentation	81.73	77.3	88.2	3	(Stamatoula Bonatsou et al., 2018)
	Candida molendinolei	table olive fermentation	73.06	68.6	80.7	7	(Stamatoula Bonatsou et al., 2018)
	Candida naeodendra	table olive fermentation	86.7	81.4	92	2	(Stamatoula Bonatsou et al., 2018)
	Candida norvegica	fermented Portugalian table olives	49.75	49.75	49.75	1	(Oliveira et al., 2017)
	Candida silvae	Greek black olives	33.25	33.25	33.25	1	(S Bonatsou et al., 2015)
	Candida tropicalis	fermented Portugalian table olives	133.7	72.65	194.76	2	(Oliveira et al., 2017)
Citeromyces	*Citeromyces matrinensis*	table olive fermentation	86.9	86.9	86.9	1	(Stamatoula Bonatsou et al., 2018)
Cystofilobasidium	*Cystofilobasidium bisporidii*	table olive fermentation	94.8	94.8	94.8	1	(Stamatoula Bonatsou et al., 2018)
Debaryomyces	*Debaryomyces hansenii*	fermented Portugalian table olives	40.71	40.71	40.71	1	(Oliveira et al., 2017)
		Greek black olives	90.37	90.37	90.37	1	(S Bonatsou et al., 2015)
Metschnikowia	*Metschnikowia pulcherrima*	Greek black olives	75.01	75.01	75.01	1	(S Bonatsou et al., 2015)
		table olive fermentation	96.43	95.7	97.5	3	(Stamatoula Bonatsou et al., 2018)
Nakazawaea	*Nakazawaea molendini-olei*	fermented black olive	15.43	15.43	15.43	1	(Porru et al., 2018)
		Olive Oil	21.11	3.8	38.41	2	(Zullo & Ciafardini, 2019)
	Nakazawaea wickerhamii	Olive Oil	75.86	75.86	75.86	1	(Zullo & Ciafardini, 2019)
Pichia	*Pichia guilliermondii*	fermented Portugalian table olives	179.43	179.43	179.43	1	(Oliveira et al., 2017)
		Greek black olives	492.05	492.05	492.05	1	(S Bonatsou et al., 2015)
		table olive fermentation	93.85	91.3	96.4	2	(Stamatoula Bonatsou et al., 2018)
	Pichia kluyveri	Greek black olives	87.77	87.77	87.77	1	(S Bonatsou et al., 2015)
		table olive fermentation	83.5	78.7	88.3	2	(Stamatoula Bonatsou et al., 2018)
	Pichia manshurica	fermented Portugalian table olives	29.63	29.63	29.63	1	(Oliveira et al., 2017)
		Greek black olives	76.96	76.96	76.96	1	(S Bonatsou et al., 2015)
		table olive fermentation	91.87	90	92.9	3	(Stamatoula Bonatsou et al., 2018)
	Pichia membranifaciens	Greek black olives	60.44	60.44	60.44	1	(S Bonatsou et al., 2015)

Rhodotorula	*Rhodotorula diobovatum*	Greek black olives	47.54	47.54	47.54	1	(S Bonatsou et al., 2015)
	Rhodotorula glutinis	table olive fermentation	86.37	75.7	91.7	3	(Stamatoula Bonatsou et al., 2018)
	Rhodotorula mucilaginosa	Greek black olives	31.33	31.33	31.33	1	(S Bonatsou et al., 2015)
		table olive fermentation	82.7	82.7	82.7	1	(Stamatoula Bonatsou et al., 2018)
Saccharomyces	*Saccharomyces boulardii*	fermented black olive	95.88	95.88	95.88	1	(Porru et al., 2018)
		fermented Portugalian table olives	44.43	44.43	44.43	1	(Oliveira et al., 2017)
		Olive Oil	66.06	66.06	66.06	1	(Zullo & Ciafardini, 2019)
	Saccharomyces cerevisiae	fermented black olive	79.68	79.68	79.68	1	(Porru et al., 2018)
		fermented Portugalian table olives	21.16	3.16	39.15	2	(Oliveira et al., 2017)
		Greek black olives	51.02	51.02	51.02	1	(S Bonatsou et al., 2015)
		table olive fermentation	93.03	89.9	98.8	6	(Stamatoula Bonatsou et al., 2018)
Wickerhamomyces	*Wickerhamomyces anomalus*	fermented black olive	49.15	49.15	49.15	1	(Porru et al., 2018)
		Greek black olives	40.48	40.48	40.48	1	(S Bonatsou et al., 2015)
		Olive Oil	33.95	17.78	50.12	2	(Zullo & Ciafardini, 2019)
Yamadazyma	*Yamadazyma terventina*	Olive Oil	17.91	10.12	25.7	2	(Zullo & Ciafardini, 2019)
Zygoascus	*Zygoascus hellenicus*	table olive fermentation	93.87	91.2	97.7	3	(Stamatoula Bonatsou et al., 2018)
Zygosaccharomyces	*Zygosaccharomyces mrakii*	fermented black olive	19.54	19.54	19.54	1	(Porru et al., 2018)

pH.2, pepsin 0.0133g/L, 2.5 h, 37°C, buffer: NaCl (2.05 g/L), KH_2PO_4 (0.60 g/ L), $CaCl_2$ (0.11 g/L) and KCl (0.37 g/L)

Table S2. The survivability of selected yeast strains undergo in-vitro intestinal conditions

Genus	Species	Sources	Survival	Minimum	Maximum	Count	Reference
Aureobasidium	*Aureobasidium pullulans*	Greek black olives	0	0	0	1	(S Bonatsou et al., 2015)
		table olive fermentation	40.85	0	91.4	6	(Stamatoula Bonatsou et al., 2018)
Candida	*Candida adriatica*	Olive Oil	7.26	6.92	7.59	2	(Zullo & Ciafardini, 2019)
	Candida boidinii	fermented black olive	12.82	3.53	22.1	2	(Porru et al., 2018)
		fermented Portugalian table olives	18.17	18.17	18.17	1	(Oliveira et al., 2017)
		table olive fermentation	60.47	0	81.6	6	(Stamatoula Bonatsou et al., 2018)
	Candida diddensiae	fermented black olive	31.63	24.76	38.49	2	(Porru et al., 2018)
		Olive Oil	0.45	0.28	0.62	2	(Zullo & Ciafardini, 2019)
		table olive fermentation	66.67	65.4	69	3	(Stamatoula Bonatsou et al., 2018)
	Candida molendinolei	table olive fermentation	62.63	51	89.8	7	(Stamatoula Bonatsou et al., 2018)
	Candida naeodendra	table olive fermentation	35.85	0	71.7	2	(Stamatoula Bonatsou et al., 2018)
	Candida norvegica	fermented Portugalian table olives	125.75	125.75	125.75	1	(Oliveira et al., 2017)
	Candida silvae	Greek black olives	60.22	60.22	60.22	1	(S Bonatsou et al., 2015)
	Candida tropicalis	fermented Portugalian table olives	58.76	56.11	61.41	2	(Oliveira et al., 2017)
Citeromyces	*Citeromyces matrinensis*	table olive fermentation	55.3	55.3	55.3	1	(Stamatoula Bonatsou et al., 2018)
Cystofilobasidium	*Cystofilobasidium bisporidii*	table olive fermentation	87.5	87.5	87.5	1	(Stamatoula Bonatsou et al., 2018)
Debaryomyces	*Debaryomyces hansenii*	fermented Portugalian table olives	97.74	97.74	97.74	1	(Oliveira et al., 2017)
		Greek black olives	0.08	0.08	0.08	1	(S Bonatsou et al., 2015)
Metschnikowia	*Metschnikowia pulcherrima*	Greek black olives	12.1	12.1	12.1	1	(S Bonatsou et al., 2015)
		table olive fermentation	89.4	87.7	91.2	3	(Stamatoula Bonatsou et al., 2018)
Nakazawaea	*Nakazawaea molendini-olei*	fermented black olive	0.02	0.02	0.02	1	(Porru et al., 2018)
		Olive Oil	0.28	0.25	0.31	2	(Zullo & Ciafardini, 2019)
	Nakazawaea wickerhamii	Olive Oil	2.34	2.34	2.34	1	(Zullo & Ciafardini, 2019)
Pichia	*Pichia guilliermondii*	fermented Portugalian table olives	68.91	68.91	68.91	1	(Oliveira et al., 2017)
		Greek black olives	63.8	63.8	63.8	1	(S Bonatsou et al., 2015)
		table olive fermentation	81.85	81.2	82.5	2	(Stamatoula Bonatsou et al., 2018)
	Pichia kluyveri	Greek black olives	40.84	40.84	40.84	1	(S Bonatsou et al., 2015)
		table olive fermentation	72.25	64.4	80.1	2	(Stamatoula Bonatsou et al., 2018)
	Pichia manshurica	fermented Portugalian table olives	11.05	11.05	11.05	1	(Oliveira et al., 2017)
		Greek black olives	57.28	57.28	57.28	1	(S Bonatsou et al., 2015)

		table olive fermentation	79.87	78.4	82.5	3	(Stamatoula Bonatsou et al., 2018)
	Pichia membranifaciens	Greek black olives	11.29	11.29	11.29	1	(S Bonatsou et al., 2015)
Rhodotorula	*Rhodotorula diobovatum*	Greek black olives	3.83	3.83	3.83	1	(S Bonatsou et al., 2015)
	Rhodotorula glutinis	table olive fermentation	70.6	62.4	76.8	3	(Stamatoula Bonatsou et al., 2018)
	Rhodotorula mucilaginosa	Greek black olives	53.38	53.38	53.38	1	(S Bonatsou et al., 2015)
		table olive fermentation	71.1	71.1	71.1	1	(Stamatoula Bonatsou et al., 2018)
Saccharomyces	*Saccharomyces boulardii*	fermented black olive	10.99	10.99	10.99	1	(Porru et al., 2018)
		fermented Portugalian table olives	99.79	99.79	99.79	1	(Oliveira et al., 2017)
		Olive Oil	6.92	6.92	6.92	1	(Zullo & Ciafardini, 2019)
	Saccharomyces cerevisiae	fermented black olive	46.6	46.6	46.6	1	(Porru et al., 2018)
		fermented Portugalian table olives	94.29	12.61	175.98	2	(Oliveira et al., 2017)
		Greek black olives	64.28	64.28	64.28	1	(S Bonatsou et al., 2015)
		table olive fermentation	54.42	6.7	90.9	6	(Stamatoula Bonatsou et al., 2018)
Wickerhamomyces	*Wickerhamomyces anomalus*	fermented black olive	5.28	5.28	5.28	1	(Porru et al., 2018)
		Greek black olives	13.39	13.39	13.39	1	(S Bonatsou et al., 2015)
		Olive Oil	0.21	0.08	0.35	2	(Zullo & Ciafardini, 2019)
Yamadazyma	*Yamadazyma terventina*	Olive Oil	1.93	1.41	2.45	2	(Zullo & Ciafardini, 2019)
Zygoascus	*Zygoascus hellenicus*	table olive fermentation	72.4	65.1	86.5	3	(Stamatoula Bonatsou et al., 2018)
Zygosaccharomyces	*Zygosaccharomyces mrakii*	fermented black olive	0.1	0.1	0.1	1	(Porru et al., 2018)

pH 8.0, pancreatin 0.1 g/L, bile salts 3.0g/L, 3.5h, 37°C) Buffer:50.81 g/L of sodium phosphate dibasic heptahydrate and 8.5 g/L of NaCl

Table S3. Evaluated of NIC (non-inhibitory concentration) and MIC (minimum inhibitory concentration) values (g/L) measured under salt (NaCl) for the yeast strains

Genus	Species	Source	NaCl%	pH	Time	Temp.	NIC (g/L) mean	Minimum	Maximum	MIC (g/L)mean	Minimum	Maximum	Count	Reference
Aureobasidium	*Aureobasidium pullulans*	cv. Kalamata table olive fermentation	0-250 g/L	3.5	12h for 7days	Not mentined	24.6	0	42.4	115.5	0	207.2	6	(Stamatoula Bonatsou et al., 2018)
				5	12h for 7days	Not mentined	49.98	41.8	71.2	162.97	110.6	191.5	6	(Stamatoula Bonatsou et al., 2018)
				6.5	12h for 7days	Not mentined	49.58	17.4	69.4	131.5	94.7	168.6	6	(Stamatoula Bonatsou et al., 2018)
		Greek black olives	0-250 g/L	Not mentined	2h	Not mentined	44.47	44.47	44.47	127.09	127.09	127.09	1	(S Bonatsou et al., 2015)
Candida	*Candida boidinii*	cv. Kalamata table olive fermentation	0-250 g/L	3.5	12h for 7days	Not mentined	52.85	26.8	74.9	103.8	89.8	117.3	6	(Stamatoula Bonatsou et al., 2018)
				5	12h for 7days	Not mentined	73.07	54.7	82.3	120.62	103.9	168.5	6	(Stamatoula Bonatsou et al., 2018)
				6.5	12h for 7days	Not mentined	70.22	58.8	82.1	113	88.6	130.2	6	(Stamatoula Bonatsou et al., 2018)
		fermented black olive	0-180 g/L	4.5	7days	28	68.48	68.48	68.48	110.11	110.11	110.11	1	(Porru et al., 2018)
	Candida diddensiae	cv. Kalamata table olive fermentation	0-250 g/L	3.5	12h for 7days	Not mentined	48.77	0	92.4	81.37	0	155.2	3	(Stamatoula Bonatsou et al., 2018)
				5	12h for 7days	Not mentined	24.97	0	74.9	32.7	0	98.1	3	(Stamatoula Bonatsou et al., 2018)
				6.5	12h for 7days	Not mentined	19	0	57	29.53	0	88.6	3	(Stamatoula Bonatsou

														et al., 2018)
		fermented black olive	0-180 g/L	4.5	7days	28	79.13	64.58	93.67	119.54	98.95	140.12	2	(Porru et al., 2018)
	Candida molendinolei	cv. Kalamata table olive fermentation	0-250 g/L	3.5	12h for 7days	Not mentined	47.11	38.8	52.7	127.57	123.4	140.9	7	(Stamatoula Bonatsou et al., 2018)
				5	12h for 7days	Not mentined	76.94	61.1	92.6	134.14	125.5	163.8	7	(Stamatoula Bonatsou et al., 2018)
				6.5	12h for 7days	Not mentined	59.31	38.3	98.1	128	113.3	154.7	7	(Stamatoula Bonatsou et al., 2018)
	Candida naeodendra	cv. Kalamata table olive fermentation	0-250 g/L	3.5	12h for 7days	Not mentined	49.7	24.3	75.1	512.35	117.7	907	2	(Stamatoula Bonatsou et al., 2018)
				5	12h for 7days	Not mentined	60.65	41.3	80	158.8	112.6	205	2	(Stamatoula Bonatsou et al., 2018)
				6.5	12h for 7days	Not mentined	81	76.6	85.4	111.85	101.3	122.4	2	(Stamatoula Bonatsou et al., 2018)
	Candida silvae	Greek black olives	0-250 g/L	Not mentined	2h	Not mentined	45.82	45.82	45.82	134.66	134.66	134.66	1	(S Bonatsou et al., 2015)
	Saccharomyces boidinii	fermented black olive	0-180 g/L	4.5	7days	28	80.94	80.94	80.94	115.43	115.43	115.43	1	(Porru et al., 2018)
Citeromyces	*Citeromyces matrinensis*	cv. Kalamata table olive fermentation	0-250 g/L	3.5	12h for 7days	Not mentined	43.4	43.4	43.4	129.8	129.8	129.8	1	(Stamatoula Bonatsou et al., 2018)
				5	12h for 7days	Not mentined	0	0	0	0	0	0	1	(Stamatoula Bonatsou et al., 2018)
				6.5	12h for 7days	Not mentined	0	0	0	0	0	0	1	(Stamatoula Bonatsou

														et al., 2018)
Cystofilobasidium	*Cystofilobasidium bisporidii*	cv. Kalamata table olive fermentation	0-250 g/L	3.5	12h for 7days	Not mentined	17	17	17	88.8	88.8	88.8	1	(Stamatoula Bonatsou et al., 2018)
				5	12h for 7days	Not mentined	7.1	7.1	7.1	165.9	165.9	165.9	1	(Stamatoula Bonatsou et al., 2018)
				6.5	12h for 7days	Not mentined	4.8	4.8	4.8	205.3	205.3	205.3	1	(Stamatoula Bonatsou et al., 2018)
Debaryomyces	*Debaryomyces hansenii*	Greek black olives	0-250 g/L	Not mentined	2h	Not mentined	50.22	50.22	50.22	153.59	153.59	153.59	1	(S Bonatsou et al., 2015)
Metschnikowia	*Metschnikowia pulcherrima*	cv. Kalamata table olive fermentation	0-250 g/L	3.5	12h for 7days	Not mentined	22.73	0	43.7	66.47	0	130.3	3	(Stamatoula Bonatsou et al., 2018)
				5	12h for 7days	Not mentined	40.2	26.4	58.1	142.3	98.4	195.7	3	(Stamatoula Bonatsou et al., 2018)
				6.5	12h for 7days	Not mentined	20.7	0	34.2	76.43	0	133.4	3	(Stamatoula Bonatsou et al., 2018)
		Greek black olives	0-250 g/L	Not mentined	2h	Not mentined	61.08	61.08	61.08	176.65	176.65	176.65	1	(S Bonatsou et al., 2015)
Nakazawaea	*Nakazawaea molendini-olei*	fermented black olive	0-180 g/L	4.5	7days	28	67.2	67.2	67.2	118.92	118.92	118.92	1	(Porru et al., 2018)
Pichia	*Pichia guilliermondii*	cv. Kalamata table olive fermentation	0-250 g/L	3.5	12h for 7days	Not mentined	24.2	0	48.4	82.85	0	165.7	2	(Stamatoula Bonatsou et al., 2018)
				5	12h for 7days	Not mentined	81.55	75.8	87.3	207.5	204.8	210.2	2	(Stamatoula Bonatsou et al., 2018)
				6.5	12h for 7days	Not mentined	62.45	61.7	63.2	226.85	174.8	278.9	2	(Stamatoula

														Bonatsou et al., 2018)
		Greek black olives	0-250 g/L	Not mentined	2h	Not mentined	109.93	109.93	109.93	261.6	261.6	261.6	1	(S Bonatsou et al., 2015)
	Pichia kluyveri	cv. Kalamata table olive fermentation	0-250 g/L	3.5	12h for 7days	Not mentined	86.5	70.9	102.1	156.05	149.4	162.7	2	(Stamatoula Bonatsou et al., 2018)
				5	12h for 7days	Not mentined	0	0	0	0	0	0	2	(Stamatoula Bonatsou et al., 2018)
				6.5	12h for 7days	Not mentined	0	0	0	0	0	0	2	(Stamatoula Bonatsou et al., 2018)
		Greek black olives	0-250 g/L	Not mentined	2h	Not mentined	84.69	84.69	84.69	209.66	209.66	209.66	1	(S Bonatsou et al., 2015)
	Pichia manshurica	cv. Kalamata table olive fermentation	0-250 g/L	3.5	12h for 7days	Not mentined	48.5	39.9	62	159.57	149.9	178.3	3	(Stamatoula Bonatsou et al., 2018)
				5	12h for 7days	Not mentined	60.67	50.2	70.5	134.37	128.4	144.5	3	(Stamatoula Bonatsou et al., 2018)
				6.5	12h for 7days	Not mentined	70	64	80.6	125.93	115.2	143.3	3	(Stamatoula Bonatsou et al., 2018)
		Greek black olives	0-250 g/L	Not mentined	2h	Not mentined	71.16	71.16	71.16	152.13	152.13	152.13	1	(S Bonatsou et al., 2015)
	Pichia membranifaciens	Greek black olives	0-250 g/L	Not mentined	2h	Not mentined	20.15	20.15	20.15	154.41	154.41	154.41	1	(S Bonatsou et al., 2015)
Rhodotorula	*Rhodotorula diobovatum*	Greek black olives	0-250 g/L	Not mentined	2h	Not mentined	49.97	49.97	49.97	123.7	123.7	123.7	1	(S Bonatsou et al., 2015)

	Rhodotorula glutinis	cv. Kalamata table olive fermentation	0-250 g/L	3.5	12h for 7days	Not mentined	27.1	16.9	35.9	99.33	80.2	116.7	3	(Stamatoula Bonatsou et al., 2018)
				5	12h for 7days	Not mentined	26.73	14.3	41.3	131.8	74.8	183.4	3	(Stamatoula Bonatsou et al., 2018)
				6.5	12h for 7days	Not mentined	30.7	11.7	49.9	211.47	99.6	404.2	3	(Stamatoula Bonatsou et al., 2018)
	Rhodotorula mucilaginosa	cv. Kalamata table olive fermentation	0-250 g/L	3.5	12h for 7days	Not mentined	52.4	52.4	52.4	95.6	95.6	95.6	1	(Stamatoula Bonatsou et al., 2018)
				5	12h for 7days	Not mentined	52.2	52.2	52.2	122.6	122.6	122.6	1	(Stamatoula Bonatsou et al., 2018)
				6.5	12h for 7days	Not mentined	62.5	62.5	62.5	119.1	119.1	119.1	1	(Stamatoula Bonatsou et al., 2018)
		Greek black olives	0-250 g/L	Not mentined	2h	Not mentined	58.26	58.26	58.26	266.02	266.02	266.02	1	(S Bonatsou et al., 2015)
Saccharomyces	*Saccharomyces boulardii*	fermented black olive	0-180 g/L	4.5	7days	28	24.54	24.54	24.54	86.39	86.39	86.39	1	(Porru et al., 2018)
	Saccharomyces cerevisiae	cv. Kalamata table olive fermentation	0-250 g/L	3.5	12h for 7days	Not mentined	35.5	21.8	45.9	146.73	119	200	6	(Stamatoula Bonatsou et al., 2018)
				5	12h for 7days	Not mentined	60.37	41.1	85.3	174.83	139.1	195.2	6	(Stamatoula Bonatsou et al., 2018)
				6.5	12h for 7days	Not mentined	40.03	0	67.1	143.8	0	214.9	6	(Stamatoula Bonatsou et al., 2018)
		fermented black olive	0-180 g/L	4.5	7days	28	24.82	24.82	24.82	86.29	86.29	86.29	1	(Porru et al., 2018)

		Greek black olives	0-250 g/L	Not mentined	2h	Not mentined	86.3	86.3	86.3	274.44	274.44	274.44	1	(S Bonatsou et al., 2015)
Wickerhamomyces	*Wickerhamomyces anomalus*	fermented black olive	0-180 g/L	4.5	7days	28	114.8	114.8	114.8	171.47	171.47	171.47	1	(Porru et al., 2018)
		Greek black olives	0-250 g/L	Not mentined	2h	Not mentined	47.27	47.27	47.27	163.18	163.18	163.18	1	(S Bonatsou et al., 2015)
Zygoascus	*Zygoascus hellenicus*	cv. Kalamata table olive fermentation	0-250 g/L	3.5	12h for 7days	Not mentined	76.53	37.7	96.4	125.23	115.4	143.2	3	(Stamatoula Bonatsou et al., 2018)
				5	12h for 7days	Not mentined	81.23	68.8	93.7	147.13	146.6	147.9	3	(Stamatoula Bonatsou et al., 2018)
				6.5	12h for 7days	Not mentined	54.43	28.3	78.3	129.63	112.9	157.9	3	(Stamatoula Bonatsou et al., 2018)
Zygosaccharomyces	*Zygosaccharomyces mrakii*	fermented black olive	0-180 g/L	4.5	7days	28	40.55	40.55	40.55	96.74	96.74	96.74	1	(Porru et al., 2018)

Media for all: YM broth

Table S4. The percentage auto-aggregation capacity of the selected yeast strains, incubated at 37C and measured at different time intervals

Genus	Species	Source	Time	Media	Mean	Autoag. minimum	Maximum	Count	Reference
Aureobasidium	*Aureobasidium pullulans*	cv. Kalamata table olive fermentation	24h	PBS	91.25	84	95	4	(Stamatoula Bonatsou et al., 2018)
			2h	PBS	84.75	76	94	4	(Stamatoula Bonatsou et al., 2018)
			4h	PBS	84	80	88	4	(Stamatoula Bonatsou et al., 2018)
Candida	*Candida adriatica*	Olive Oil	4h	Sodium Phosphate buffer	21	10	32	2	(Zullo & Ciafardini, 2019)
	Candida boidinii	cv. Kalamata table olive fermentation	24h	PBS	93.5	90	96	4	(Stamatoula Bonatsou et al., 2018)
			2h	PBS	77	58	90	4	(Stamatoula Bonatsou et al., 2018)
			4h	PBS	74.25	57	84	4	(Stamatoula Bonatsou et al., 2018)
		fermented Portugalian table olives	24h	0.9%NaCl salin solution	50.97	25.36	76.58	2	(Oliveira et al., 2017)
			2h	0.9%NaCl salin solution	17.48	8.96	26	2	(Oliveira et al., 2017)
			4h	0.9%NaCl salin solution	28	14	42	2	(Oliveira et al., 2017)
	Candida Caribbica	pineapple peel	24h	PBS	99	99	99	2	(Amorim et al., 2018)
	Candida diddensiae	cv. Kalamata table olive fermentation	24h	PBS	92	92	92	2	(Stamatoula Bonatsou et al., 2018)
			2h	PBS	60	60	60	2	(Stamatoula Bonatsou et al., 2018)
			4h	PBS	64	64	64	2	(Stamatoula Bonatsou et al., 2018)
		Olive Oil	4h	Sodium Phosphate buffer	35.5	23	48	2	(Zullo & Ciafardini, 2019)
	Candida Lusitaniae	pineapple peel	24h	PBS	96	96	96	3	(Amorim et al., 2018)
			2h	PBS	4	0	7	3	(Amorim, Piccoli, and Duarte 2018)
			4h	PBS	40.67	38	43	3	(Amorim et al., 2018)
	Candida molendinolei	cv. Kalamata table olive fermentation	24h	PBS	92.67	90	96	3	(Stamatoula Bonatsou et al., 2018)
			2h	PBS	42.33	22	75	3	(Stamatoula Bonatsou et al., 2018)
			4h	PBS	48.33	30	78	3	(Stamatoula Bonatsou et al., 2018)
	Candida naeodendra	cv. Kalamata table olive fermentation	24h	PBS	94	94	94	1	(Stamatoula Bonatsou et al., 2018)
			2h	PBS	83	83	83	1	(Stamatoula Bonatsou et al., 2018)
			4h	PBS	79	79	79	1	(Stamatoula Bonatsou et al., 2018)
	Candida norvegica	fermented Portugalian table olives	24h	0.9%NaCl salin solution	83	83	83	1	(Oliveira et al., 2017)
			2h	0.9%NaCl salin solution	38.27	38.27	38.27	1	(Oliveira et al., 2017)
			4h	0.9%NaCl salin solution	59	59	59	1	(Oliveira et al., 2017)
	Candida orthopsilosis	fermented food, cocoa fermentation, and kefi	5h	PBS	87.5	87	88	2	(Menezes et al., 2020)

	Candida pararugosa	food origin	30min	0.9%NaCl salin solution	63	63	63	1	(Fernandez-Pacheco Rodríguez et al., 2018
	Candida quercitrusa	fermented food, cocoa fermentation, and kefi	5h	PBS	96	96	96	1	(Menezes et al., 2020)
	Candida sake	food origin	30min	0.9%NaCl salin solution	16	16	16	1	(Fernandez-Pacheco Rodríguez et al., 2018
	Candida tropicalis	fermented Portugalian table olives	24h	0.9%NaCl salin solution	86.16	78.37	93.94	2	(Oliveira et al., 2017)
			2h	0.9%NaCl salin solution	70.5	66	75	2	(Oliveira et al., 2017)
			4h	0.9%NaCl salin solution	74.42	71.29	77.54	2	(Oliveira et al., 2017)
	Candida vini	food origin	30min	0.9%NaCl salin solution	42	42	42	1	(Fernandez-Pacheco Rodríguez et al., 2018
Cystofilobasidium	*Cystofilobasidium bisporidii*	cv. Kalamata table olive fermentation	24h	PBS	90	90	90	1	(Stamatoula Bonatsou et al., 2018)
			2h	PBS	37	37	37	1	(Stamatoula Bonatsou et al., 2018)
			4h	PBS	36	36	36	1	(Stamatoula Bonatsou et al., 2018)
Debaryomyce	*Debaryomyces hansenii*	fermented Portugalian table olives	24h	0.9%NaCl salin solution	34.37	34.37	34.37	1	(Oliveira et al., 2017)
			2h	0.9%NaCl salin solution	31	31	31	1	(Oliveira et al., 2017)
			4h	0.9%NaCl salin solution	30.83	30.83	30.83	1	(Oliveira et al., 2017)
Galactomyces	*Galactomyces reessii*	fermented Portugalian table olives	24h	0.9%NaCl salin solution	45.33	31	70.5	3	(Oliveira et al., 2017)
			2h	0.9%NaCl salin solution	28.44	22.01	34.86	2	(Oliveira et al., 2017)
			4h	0.9%NaCl salin solution	29.21	29.21	29.21	1	(Oliveira et al., 2017)
Hanseniaspora	*Hanseniaspora opuntiae*	fermented food, cocoa fermentation, and kefi	5h	PBS	66	66	66	1	(Menezes et al., 2020)
	Hanseniaspora osmophila	food origin	30min	0.9%NaCl salin solution	48	32	64	2	(Fernandez-Pacheco Rodríguez et al., 2018
	Hanseniaspora uvarum	fermented food, cocoa fermentation, and kefi	5h	PBS	85	84	86	2	(Menezes et al., 2020)
Kluyveromyces	*Kluyveromyces lactis*	traditional kefir grains	2h	PBS	35	35	35	1	(Gut et al., 2019)
	Kluyveromyces marxianus	fermented food, cocoa fermentation, and kefi	5h	PBS	83	76	90	2	(Menezes et al., 2020)
	Kluyveromyces thermotolerans	food origin	30min	0.9%NaCl salin solution	28.5	14	43	2	(Fernandez-Pacheco Rodríguez et al., 2018
Metschnikowia	*Metschnikowia pulcherrima*	cv. Kalamata table olive fermentation	24h	PBS	90	85	94	3	(Stamatoula Bonatsou et al., 2018)
			2h	PBS	62.33	28	80	3	(Stamatoula Bonatsou et al., 2018)
			4h	PBS	66	31	84	3	(Stamatoula Bonatsou et al., 2018)
		food origin	30min	0.9%NaCl salin solution	13	13	13	1	(Fernandez-Pacheco Rodríguez et al., 2018
Meyerozyma	*Meyerozyma Caribbica*	pineapple peel	2h	PBS	14.5	14	15	2	(Amorim et al., 2018)
			4h	PBS	40.5	36	45	2	(Amorim et al., 2018)
Nakazawaea	*Nakazawaea molendini-olei*	Olive Oil	4h	Sodium Phosphate buffer	18	8	28	2	(Zullo & Ciafardini, 2019)
	Nakazawaea wickerhamii	Olive Oil	4h	Sodium Phosphate buffer	18	18	18	1	(Zullo & Ciafardini, 2019)
Ogataea	*Ogataea polymorpha*	food origin	30min	0.9%NaCl salin solution	4	4	4	1	(Fernandez-Pacheco Rodríguez et al., 2018

Pichia	*Pichia membranifaciens*	fermented Portugalian table olives	24h	0.9%NaCl salin solution	76.6	76.13	77.08	2	(Oliveira et al., 2017)
	Pichia anomala	food origin	30min	0.9%NaCl salin solution	19.5	11	28	2	(Fernandez-Pacheco Rodríguez et al., 2018
	Pichia caribbica	food origin	30min	0.9%NaCl salin solution	7	7	7	1	(Fernandez-Pacheco Rodríguez et al., 2018
	Pichia galeiforms	food origin	30min	0.9%NaCl salin solution	12	12	12	1	(Fernandez-Pacheco Rodríguez et al., 2018
	Pichia guilliermondii	fermented food, cocoa fermentation, and kefi	5h	PBS	83	83	83	1	(Menezes et al., 2020)
		fermented Portugalian table olives	24h	0.9%NaCl salin solution	73.66	73.66	73.66	1	(Oliveira et al., 2017)
			2h	0.9%NaCl salin solution	25	25	25	1	(Oliveira et al., 2017)
			4h	0.9%NaCl salin solution	30.66	30.66	30.66	1	(Oliveira et al., 2017)
	Pichia kluyveri	cv. Kalamata table olive fermentation	24h	PBS	90	88	92	2	(Stamatoula Bonatsou et al., 2018)
			2h	PBS	45	41	49	2	(Stamatoula Bonatsou et al., 2018)
			4h	PBS	53	48	58	2	(Stamatoula Bonatsou et al., 2018)
		fermented food, cocoa fermentation, and kefi	5h	PBS	92	92	92	1	(Menezes et al., 2020)
		food origin	30min	0.9%NaCl salin solution	30	30	30	1	(Fernandez-Pacheco Rodríguez et al., 2018
	Pichia kudriavzevii	food origin	30min	0.9%NaCl salin solution	16.5	5	28	2	(Fernandez-Pacheco Rodríguez et al., 2018
	Pichia manshurica	cv. Kalamata table olive fermentation	24h	PBS	91	89	92	3	(Stamatoula Bonatsou et al., 2018)
			2h	PBS	77	61	88	3	(Stamatoula Bonatsou et al., 2018)
			4h	PBS	75	55	89	3	(Stamatoula Bonatsou et al., 2018)
		fermented Portugalian table olives	24h	0.9%NaCl salin solution	76.86	76.86	76.86	1	(Oliveira et al., 2017)
			2h	0.9%NaCl salin solution	13	13	13	1	(Oliveira et al., 2017)
			4h	0.9%NaCl salin solution	24.75	24.75	24.75	1	(Oliveira et al., 2017)
	Pichia membranifaciens	fermented food, cocoa fermentation, and kefi	5h	PBS	93	93	93	1	(Menezes et al., 2020)
		fermented Portugalian table olives	2h	0.9%NaCl salin solution	51.58	48.16	55	2	(Oliveira et al., 2017)
			4h	0.9%NaCl salin solution	56.5	47	66	2	(Oliveira et al., 2017)
		food origin	30min	0.9%NaCl salin solution	41	41	41	1	(Fernandez-Pacheco Rodríguez et al., 2018
Rhodotorula	*Rhodotorula glutinis*	cv. Kalamata table olive fermentation	24h	PBS	91.67	87	96	3	(Stamatoula Bonatsou et al., 2018)
			2h	PBS	50	38	62	3	(Stamatoula Bonatsou et al., 2018)
			4h	PBS	66	46	80	3	(Stamatoula Bonatsou et al., 2018)
		fermented Portugalian table olives	24h	0.9%NaCl salin solution	23.67	23.67	23.67	1	(Oliveira et al., 2017)
			2h	0.9%NaCl salin solution	2.86	2.86	2.86	1	(Oliveira et al., 2017)
			4h	0.9%NaCl salin solution	5.12	5.12	5.12	1	(Oliveira et al., 2017)

	Rhodotorula graminis	fermented Portugalian table olives	24h	0.9%NaCl salin solution	20.11	20.11	20.11	1	(Oliveira et al., 2017)
			2h	0.9%NaCl salin solution	3.35	3.35	3.35	1	(Oliveira et al., 2017)
			4h	0.9%NaCl salin solution	4	4	4	1	(Oliveira et al., 2017)
	Rhodotorula mucilaginosa	cv. Kalamata table olive fermentation	24h	PBS	90	90	90	1	(Stamatoula Bonatsou et al., 2018)
			2h	PBS	74	74	74	1	(Stamatoula Bonatsou et al., 2018)
			4h	PBS	74	74	74	1	(Stamatoula Bonatsou et al., 2018)
Saccharomyces	*Saccharomyces boulardii*	fermented food, cocoa fermentation, and kefi	5h	PBS	96	96	96	1	(Menezes et al., 2020)
		fermented Portugalian table olives	24h	0.9%NaCl salin solution	55.9	34.75	77.05	2	(Oliveira et al., 2017)
			2h	0.9%NaCl salin solution	25.5	25.5	25.5	1	(Oliveira et al., 2017)
		Olive Oil	4h	Sodium Phosphate buffer	43	43	43	1	(Zullo & Ciafardini, 2019)
		traditional kefir grains	2h	PBS	37	31	43	2	(Gut et al., 2019)
	Saccharomyces cerevisiae	cv. Kalamata table olive fermentation	24h	PBS	95.25	94	96	4	(Stamatoula Bonatsou et al., 2018)
			2h	PBS	84.75	72	90	4	(Stamatoula Bonatsou et al., 2018)
			4h	PBS	82.5	67	91	4	(Stamatoula Bonatsou et al., 2018)
		fermented food, cocoa fermentation, and kefi	5h	PBS	94.07	68	99	14	(Menezes et al., 2020)
		fermented Portugalian table olives	24h	0.9%NaCl salin solution	83.02	74.33	91.71	2	(Oliveira et al., 2017)
			2h	0.9%NaCl salin solution	56.99	54	59.97	2	(Oliveira et al., 2017)
			4h	0.9%NaCl salin solution	71.62	71.23	72	2	(Oliveira et al., 2017)
		food origin	30min	0.9%NaCl salin solution	20	20	20	1	(Fernandez-Pacheco Rodríguez et al., 2018
	Saccharomyces cerevisiae var. boulardii	food origin	30min	0.9%NaCl salin solution	19	19	19	1	(Fernandez-Pacheco Rodríguez et al., 2018
		pineapple peel	24h	PBS	96	96	96	1	(Amorim et al., 2018)
			2h	PBS	3	3	3	1	(Amorim et al., 2018)
			4h	PBS	44	44	44	1	(Amorim et al., 2018)
	Saccharomyces unisporus	traditional kefir grains	2h	PBS	43	43	43	1	(Gut et al., 2019)
	Saccharomycescerevisiae	fermented food, cocoa fermentation, and kefi	5h	PBS	96	96	96	1	(Menezes et al., 2020)
Torulaspora	*Torulaspora delbrueckii*	food origin	30min	0.9%NaCl salin solution	19	19	19	1	(Fernandez-Pacheco Rodríguez et al., 2018
Wickerhamomyces	*Wickerhamomyces anomalus*	Olive Oil	4h	Sodium Phosphate buffer	30	28	32	2	(Zullo & Ciafardini, 2019)
Yamadazyma	*Yamadazyma terventina*	Olive Oil	4h	Sodium Phosphate buffer	31.5	30	33	2	(Zullo & Ciafardini, 2019)
Zygoascus	*Zygoascus hellenicus*	cv. Kalamata table olive fermentation	24h	PBS	90.67	88	92	3	(Stamatoula Bonatsou et al., 2018)
			2h	PBS	43	25	65	3	(Stamatoula Bonatsou et al., 2018)
			4h	PBS	51	36	70	3	(Stamatoula Bonatsou et al., 2018)

Zygosaccharomyces	*Zygosaccharomyces bailii*	food origin	30min	0.9%NaCl salin solution	35	35	35	1	(Fernandez-Pacheco Rodríguez et al., 2018
	Zygosaccharomyces fermentati	food origin	30min	0.9%NaCl salin solution	13	4	26	3	(Fernandez-Pacheco Rodríguez et al., 2018

Table S5. The percentage of hydrophobicity capacity of the selected yeast strains, incubated at 37C and measured at different time intervals.

Genus	Species	Source	Time	Temp	Media	Reference	Hexadecane				Xylene				Tolene			
							Mean	Minimum	Maximum	Count	Mean	Minimum	Maximum	Count	Mean	Minimum	Maximum	Count
Candida	Candida adriatica	Olive Oil	1h	not mentioned	PBS	(Zullo & Ciafardini, 2019)	37.5	19.5	55.5	2	.	.	.	2	.	.	.	2
	Candida diddensiae	Olive Oil	1h	not mentioned	PBS	(Zullo & Ciafardini, 2019)	29	16.5	41.5	2	.	.	.	2	.	.	.	2
	Candida Lusitaniae	pineapple peel	30 min	37	PBS	(Amorim et al., 2018)	91.9	91.9	91.9	1	99.66	99.66	99.66	1	.	.	.	1
	Candida orthopsilosis	Brazilian indigenous fermented food, cocoa fermentation, and kefir	1h	37	Phosphate Buffer	(Menezes et al., 2020)	98.9	97.8	100	2	.	.	.	2	.	.	.	2
	Candida pararugosa	food origin	1h	37	KNO3 solution	(Fernandez-Pacheco Rodríguez et al., 2018	51.09	51.09	51.09	1	.	.	.	1	25.87	25.87	25.87	1
	Candida quercitrusa	Brazilian indigenous fermented food, cocoa fermentation, and kefir	1h	37	Phosphate Buffer	(Menezes et al., 2020)	98.9	98.9	98.9	1	.	.	.	1	.	.	.	1
	Candida sake	food origin	1h	37	KNO3 solution	(Fernandez-Pacheco Rodríguez et al., 2018	6.64	6.64	6.64	1	.	.	.	1	13.35	13.35	13.35	1
	Candida vini	food origin	1h	37	KNO3 solution	(Fernandez-Pacheco Rodríguez et al., 2018	30.81	30.81	30.81	1	.	.	.	1	33.09	33.09	33.09	1
Hanseniaspora	Hanseniaspora opuntiae	Brazilian indigenous fermented food, cocoa fermentation, and kefir	1h	37	Phosphate Buffer	(Menezes et al., 2020)	98.7	98.7	98.7	1	.	.	.	1	.	.	.	1
	Hanseniaspora osmophila	food origin	1h	37	KNO3 solution	(Fernandez-Pacheco Rodríguez et al., 2018	32.02	31.51	32.52	2	.	.	.	2	30.22	27.64	32.8	2
	Hanseniaspora uvarum	Brazilian indigenous fermented food, cocoa fermentation, and kefir	1h	37	Phosphate Buffer	(Menezes et al., 2020)	98	97.5	98.5	2	.	.	.	2	.	.	.	2
Kluyveromyces	Kluyveromyces lactis	traditional kefir grains	1h	37	PBS	(Gut et al., 2019)	88.75	88.75	88.75	1	.	.	.	1	.	.	.	1
	Kluyveromyces marxianus	Brazilian indigenous fermented food, cocoa fermentation, and kefir	1h	37	Phosphate Buffer	(Menezes et al., 2020)	99.45	98.9	100	2	.	.	.	2	.	.	.	2
	Kluyveromyces thermotolerans	food origin	1h	37	KNO3 solution	(Fernandez-Pacheco Rodríguez et al., 2018	20.77	10.41	31.12	2	.	.	.	2	14.46	3.67	25.25	2
Metschnikowia	Metschnikowia pulcherrima	food origin	1h	37	KNO3 solution	(Fernandez-Pacheco Rodríguez et al., 2018	15.22	15.22	15.22	1	.	.	.	1	15.05	15.05	15.05	1
Meyerozyma	Meyerozyma Caribbica	pineapple peel	30 min	37	PBS	(Amorim et al., 2018)	68.2	46.3	90.1	2	64.13	28.7	99.55	2	.	.	.	2
Nakazawaea	Nakazawaea molendini-olei	Olive Oil	1h	not mentioned	PBS	(Zullo & Ciafardini, 2019)	0	0	0	2	.	.	.	2	.	.	.	2
	Nakazawaea wickerhamii	Olive Oil	1h	not mentioned	PBS	(Zullo & Ciafardini, 2019)	0	0	0	1	.	.	.	1	.	.	.	1
Ogataea	Ogataea polymorpha	food origin	1h	37	KNO3 solution	(Fernandez-Pacheco Rodríguez et al., 2018	10.55	10.55	10.55	1	.	.	.	1	5.44	5.44	5.44	1
Pichia	Pichia anomala	food origin	1h	37	KNO3 solution	(Fernandez-Pacheco Rodríguez et al., 2018	27.16	23.84	30.47	2	.	.	.	2	31.71	31.45	31.97	2
	Pichia caribbica	food origin	1h	37	KNO3 solution	(Fernandez-Pacheco Rodríguez et al., 2018	3.16	3.16	3.16	1	.	.	.	1	10.76	10.76	10.76	1

	Pichia galeiforms	food origin	1h	37	KNO3 solution	(Fernandez-Pacheco Rodríguez et al., 2018	21.49	21.49	21.49	1	.	.	.	1	29.6	29.6	29.6	1
	Pichia guilliermondii	Brazilian indigenous fermented food, cocoa fermentation, and kefir	1h	37	Phosphate Buffer	(Menezes et al., 2020)	94.9	94.9	94.9	1	.	.	.	1	.	.	.	1
	Pichia kluyveri	Brazilian indigenous fermented food, cocoa fermentation, and kefir	1h	37	Phosphate Buffer	(Menezes et al., 2020)	99.3	99.3	99.3	1	.	.	.	1	.	.	.	1
		food origin	1h	37	KNO3 solution	(Fernandez-Pacheco Rodríguez et al., 2018	37.9	37.9	37.9	1	.	.	.	1	10.55	10.55	10.55	1
	Pichia kudriavzevii	food origin	1h	37	KNO3 solution	(Fernandez-Pacheco Rodríguez et al., 2018	27.54	22.09	32.99	2	.	.	.	2	11.13	11.03	11.22	2
	Pichia membranifaciens	Brazilian indigenous fermented food, cocoa fermentation, and kefir	1h	37	Phosphate Buffer	(Menezes et al., 2020)	99.7	99.7	99.7	1	.	.	.	1	.	.	.	1
		food origin	1h	37	KNO3 solution	(Fernandez-Pacheco Rodríguez et al., 2018	33.04	33.04	33.04	1	.	.	.	1	15.66	15.66	15.66	1
Saccharomyces	Saccharomyces boulardii	Brazilian indigenous fermented food, cocoa fermentation, and kefir	1h	37	Phosphate Buffer	(Menezes et al., 2020)	99.4	99.4	99.4	1	.	.	.	1	.	.	.	1
		Olive Oil	1h	not mentioned	PBS	(Zullo & Ciafardini, 2019)	26	26	26	1	.	.	.	1	.	.	.	1
		traditional kefir grains	1h	37	PBS	(Gut et al., 2019)	18.38	15.58	21.18	2	.	.	.	2	.	.	.	2
	Saccharomyces cerevisiae	Brazilian indigenous fermented food, cocoa fermentation, and kefir	1h	37	Phosphate Buffer	(Menezes et al., 2020)	99.52	96.5	100	15	.	.	.	15	.	.	.	15
		food origin	1h	37	KNO3 solution	(Fernandez-Pacheco Rodríguez et al., 2018	38.74	38.74	38.74	1	.	.	.	1	26.68	26.68	26.68	1
	Saccharomyces cerevisiae var. boulardii	food origin	1h	37	KNO3 solution	(Fernandez-Pacheco Rodríguez et al., 2018	37.15	37.15	37.15	1	.	.	.	1	32.41	32.41	32.41	1
	Saccharomyces unisporus	traditional kefir grains	1h	37	PBS	(Gut et al., 2019)	30	30	30	1	.	.	.	1	.	.	.	1
Torulaspora	Torulaspora delbrueckii	food origin	1h	37	KNO3 solution	(Fernandez-Pacheco Rodríguez et al., 2018	37.41	37.41	37.41	1	.	.	.	1	19.34	19.34	19.34	1
Wickerhamomyces	Wickerhamomyces anomalus	Olive Oil	1h	not mentioned	PBS	(Zullo & Ciafardini, 2019)	22.25	20.5	24	2	.	.	.	2	.	.	.	2
Yamadazyma	Yamadazyma terventina	Olive Oil	1h	not mentioned	PBS	(Zullo & Ciafardini, 2019)	41.5	37.5	45.5	2	.	.	.	2	.	.	.	2
Zygosaccharomyces	Zygosaccharomyces bailii	food origin	1h	37	KNO3 solution	(Fernandez-Pacheco Rodríguez et al., 2018	54.16	54.16	54.16	1	.	.	.	1	35.11	35.11	35.11	1
	Zygosaccharomyces fermentati	food origin	1h	37	KNO3 solution	(Fernandez-Pacheco Rodríguez et al., 2018	9.59	8.18	10.33	3	.	.	.	3	6.47	3.12	8.28	3
